# The *Phoebe* genome sheds light on the evolution of magnoliids

**DOI:** 10.1038/s41438-020-00368-z

**Published:** 2020-09-01

**Authors:** Shi-Pin Chen, Wei-Hong Sun, Yuan-Fang Xiong, Yu-Ting Jiang, Xue-Die Liu, Xing-Yu Liao, Di-Yang Zhang, Shu-Zhen Jiang, Yu Li, Bin Liu, Liang Ma, Xia Yu, Li He, Bao Liu, Jin-Lin Feng, Li-Zhen Feng, Zhi-Wen Wang, Shuang-Quan Zou, Si-Ren Lan, Zhong-Jian Liu

**Affiliations:** 1 College of Forestry, Fujian Agriculture and Forestry University, 350002 Fuzhou, China; 2 Key Laboratory of National Forestry and Grassland Administration for Orchid Conservation and Utilization at the College of Landscape Architecture, Fujian Agriculture and Forestry University, 350002 Fuzhou, China; 3 PubBio-Tech, 430070 Wuhan, China; 4 Zhejiang Institute of Subtropical Crops, Zhejiang Academy of Agricultural Sciences, 325005 Wenzhou, China; 5 Institute of Vegetable and Flowers, Shandong Academy of Agricultural Sciences, 250100 Jinan, China

**Keywords:** Genome, Genome evolution

## Abstract

Lauraceae includes the genus *Phoebe*, and the family is linked to the evolution of magnoliids. We sequenced the genome of *Phoebe bournei* Nanmu. The assembled genome size was 989.19 Mb, with a contig N50 value of 2.05 Mb. A total of 28,198 protein-coding genes were annotated in *P. bournei*. Whole-genome duplication (WGD) analysis showed that Lauraceae has experienced two WGD events; the older WGD event occurred just before the divergence of Lauraceae and Magnoliales, and the more recent WGD was shared by all lineages of Lauraceae. The phylogenetic tree showed that magnoliids form a sister clade to monocots and eudicots. We also identified 63 MADS-box genes, including *AGL12*-like genes that may be related to the regulation of *P. bournei* roots and *FIN219*-like genes encoding GH3 proteins, which are involved in photomorphogenesis. *SAUR50*-like genes involved in light signal-mediated pedicel or stem development were also identified. Four *ATMYB46-* and three *PtrEPSP*-homologous genes related to lignin biosynthesis were identified. These genes may be associated with the formation of straight trunks in *P. bournei*. Overall, the *P. bournei* reference genome provides insight into the origin, evolution, and diversification of *Phoebe* and other magnoliids.

## Introduction

Lauraceae belongs to Laurales, which together with Canellales, Piperales, and Magnoliales, constitute the magnoliids, including 9000 species^1–3^. The relationships among the magnoliids, eudicots, and monocots remain unclear, even after the publication of four magnoliid genomes^4–8^. The genomes of *Piper nigrum*, *Persea americana*, and *Liriodendron chinense* support the magnoliids as a sister clade of monocots and eudicots^4–6^, while the genome of stout camphor *Cinnamomum kanehirae* supports magnoliids as a sister clade of eudicots^7^. The conflicts in terms of phylogenetic tree location indicate that additional genomic data are needed to more clearly elucidate the relationships among magnoliids, eudicots, and monocots, especially the species within Lauraceae.

Lauraceae is an important economic and ecological family including 2850 species of herbs, shrubs, and trees, mainly distributed in tropical and subtropical regions of Asia and South America^9^. The genus *Phoebe* within Lauraceae includes ~100 species of evergreen trees and shrubs^10^. *Phoebe bournei* (Nanmu) is endemic to China, where it is a protected species^10^. Wood from *P. bournei*, known as “wood with golden wire”, is used in the production of high-quality furniture and handicrafts due to its vertical wood texture, unique fragrance, resistance to insects and rot, durability, and beauty^11,12^. In ancient times, *P. bournei* timber was often used in palace construction to produce columns that represented the power and status of the nobles^10,13,14^. *P. bournei* is often used as a street tree because of its straight trunk and broad crown^15^. Due to intensive deforestation, poor seed germination, slow growth, and illegal timber logging, natural populations of *P. bournei* are now fragmented and threatened^11–17^.

Here, we report a reference genome of *P. bournei* obtained using the PacBio sequencing platform. The results can help to reveal its phylogenetic position within the magnoliids. Analysis of the *P. bournei* genome will provide insights into the demographic history of magnoliids, and data for future conservation efforts and biological research.

## Results and discussion

### Genome sequencing and assembly

To completely sequence the *P. bournei* genome, a total of 102.05 Gb of raw data were generated from 500 bp-insert libraries by Illumina sequencing (Supplementary Table 1). Survey analysis indicated that the *P. bournei* genome shows a high level of heterozygosity, corresponding to 1.54% of the 1.00 Gb genome sized according to 19 *K*-mer analysis (Supplementary Fig. 1). For the de novo whole-genome sequencing of *P. bournei*, we obtained 109.83 Gb of raw data using PacBio sequencing (Supplementary Fig. 2 and Supplementary Table 2). The assembled genome was 989.19 Mb, with a contig N50 value of 2.05 Mb (Supplementary Table 3). Benchmarking Universal Single-Copy Orthologs (BUSCO)^18^ assessment showed that the completeness of the gene set of the assembled genome was 95% (Supplementary Table 3), and the Illumina read alignment rate was 98.87% (Supplementary Table 4), indicating that the *P. bournei* genome assembly was of high quality and could be used for subsequent analysis.

### Gene prediction and annotation

We annotated 28,198 protein-coding genes from the assembled *P. bournei* genome, 95.44% of which were supported by de novo and transcriptome data (Supplementary Fig. 3 and Supplementary Table 5). The proteome of the protein-coding genes of *P. bournei* was estimated to be 81.1% complete based on BUSCO analysis (Supplementary Table 6)^18^. We also identified 145 microRNAs, 813 transfer RNAs, 2417 ribosomal RNAs, and 519 small nuclear RNAs (Supplementary Table 7).

Through a combination of homology-based searches and de novo prediction, we estimated that 68.51% of the *P. bournei* genome consisted of repetitive sequences, with LTR/Gypsy sequences accounting for 25.22% of the genome and LTR/Copia sequences accounting for 13.67% (Supplementary Figs. 4, 5 and Supplementary Tables 8, 9). The *C. kanehirae* genome showed 20.5% fewer repeat sequences than the *P. bournei* genome, while the abundance of LTR/Gypsy and LTR/Copia sequences was 15.53% and 10.21% greater, respectively, than in *P. bournei*^7^. The insertion time of the LTR, Copia, and Gypsy elements in *P. bournei* was ~0.2 million years ago (Supplementary Fig. 6). We identified 27,011 genes that were annotated in seven functional databases. Among these genes, 19,417 (68.86%) were annotated with KOG terms, 19,649 (69.68%) were annotated with KEGG Ortholog terms, and 15,470 (54.86%) were annotated with Gene Ontology terms (Supplementary Table 10).

### Evolution of gene families

**Fig. 1:**
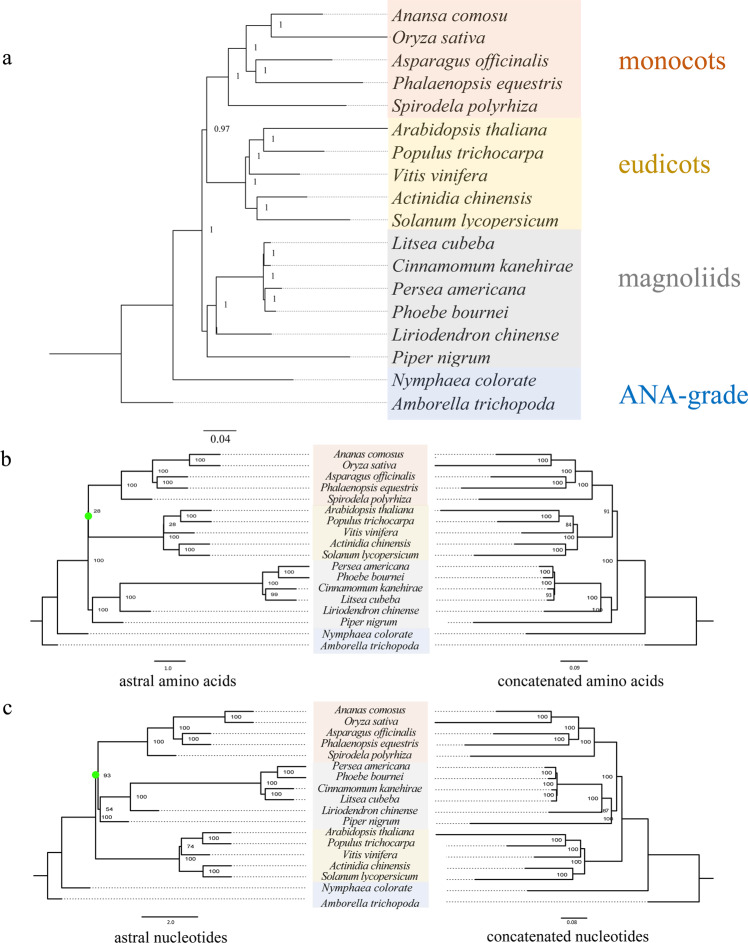
Comparison of phylogenetic trees constructed by different methods. **a** Phylogenetic tree based on the Bayesian method. **b** Phylogenetic tree based on the ASTRAL (left) and concatenated (right) amino acid sequences. **c** Phylogenetic tree based on the ASTRAL (left) and concatenated (right) nucleotide sequences

Laurales belongs to the magnoliids, and the evolutionary position of magnoliids is still unclear^4–8,19,20^. We constructed a high-confidence phylogenetic tree based on 292 single-copy gene families extracted from the genomes of 18 species (Supplementary Fig. 7 and Supplementary Table 11). The phylogenetic trees were constructed using the phase 1 loci of orthologous genes, with the branch lengths representing evolutionary rates. The phylogenetic tree showed that magnoliids, including *C. kanehirae*, *P. bournei*, *Persea americana*, *Litsea cubeba*, *P. nigrum*, and *L. chinense*, formed a sister group to the monocot−eudicot clade (Fig. 1a) based on the Bayesian method. This is consistent with the phylogenetic trees of magnoliids based on the *L. chinense*^5^ genome and the *P. americana*^6^ genome. To further determine the positional relationships of magnoliids, monocots, and eudicots, we constructed concatenated and ASTRAL trees based on protein and nucleotide sequences. The phylogenetic trees constructed via the ASTRAL and concatenated methods based on amino acids also showed that the magnoliid clade was sister to the monocot-eudicot clade (Fig. 1b; Supplementary Fig. 8). However, the phylogenetic trees constructed via the ASTRAL and concatenated methods based on nucleotides provided support for a recent common ancestor of magnoliids and monocots, which formed a clade sister to the eudicot clade (Fig. 1c). Incomplete lineage sorting is better understood as a problem of ancestral polymorphism that does not sort according to the species tree, and this polymorphism is represented in terms of haplotypes or alleles. Copy number variations can also help define haplotypic or allelic states, and copy number variants (e.g., tandem duplicates) can contribute to incomplete lineage sorting. Thus, genealogies based on orthology remain difficult to differentiate from those based on orthology plus paralogy. Therefore, we favored a phylogenetic tree based on the Bayesian method. In addition, using the MCMC tree with fossil calibration, we estimated that the Lauraceae divergence time was 127.56 Mya, and the divergence time between *P. bournei* and *P. americana* was 14.05 Mya (Supplementary Fig. 9).

**Fig. 2:**
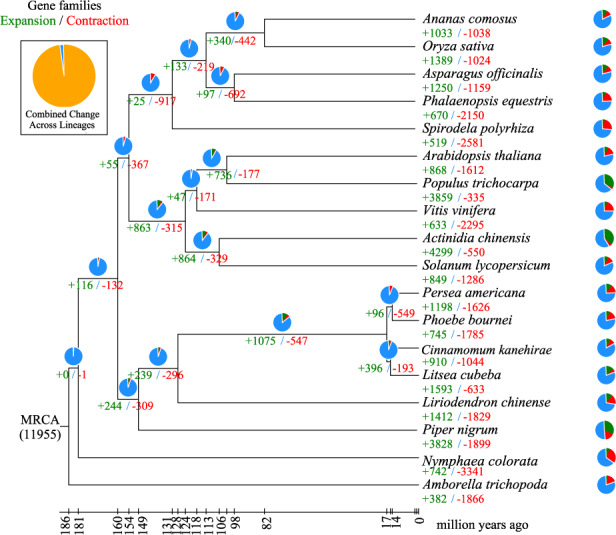
The expansion and contraction of gene families. The green numbers are the numbers of expanded gene families, and the red numbers are the numbers of contracted gene families. The blue portions of the pie charts represent the gene families whose copy numbers are constant. The orange portions of the pie charts represent the proportion of 11,968 gene families found in the most recent common ancestor (MRCA) that have expanded or contracted during recent differentiation

The expansion and contraction of orthologous gene families were determined based on a probabilistic graphical model (Fig. 2). A total of 1075 gene families were expanded in the lineage leading to Laurales, and 547 families were contracted. A total of 745 gene families were expanded in *P. bournei*, compared with 1198 and 910 in *P. americana* and *C. kanehirae*, respectively. At the same time, 1785 gene families were contracted in *P. bournei*, compared with 1626 and 1044 in *P. americana* and *C. kanehirae*, respectively.

### Synteny analysis and whole-genome duplications (WGD)

**Fig. 3:**
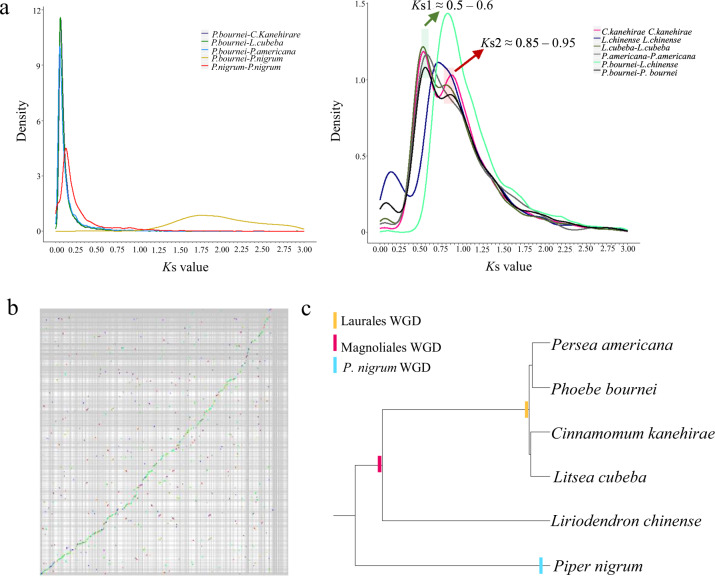
WGD analysis. **a** *K*s distribution in *P. bournei* and other species. Peaks in the intraspecies *K*s distribution indicate whole-genome polyploidization events, and peaks in the interspecies *K*s distribution indicate speciation events. **b** Collinear point diagram of the *P. bournei* genome. There are multiple 1:3 collinear regions in the *P. bournei* genome, among which one extra green copy region provides evidence of recent WGD, and two extra red copy regions provide evidence of ancient WGD events. **c** The phylogeny of magnoliids with WGD events

The distribution of *K*s values in the *P. bournei*, *C. kanehirae*, *P. americana*, and *L. cubeba* genomes showed two clear peaks, one at *K*s1 ≈ 0.5–0.6 and the other at *K*s2 ≈ 0.85–0.95 (Fig. 3a). These two *K*s peaks were greater than the *K*s values of the differentiation peaks of *P. bournei*–*C. kanehirae*, *P. bournei*–*L. cubeba*, and *P. bournei*–*P. americana* (*K*s < 0.1). This result indicated that the common ancestor of Lauraceae (*P. bournei*, *C. kanehirae*, *L. cubeba*, and *P. americana*) underwent two polyploidization events before the groups diverged. Collinearity analysis confirmed that these two polyploidy events of *P. bournei* were WGD events (Fig. 3b). *C. kanehirae*, *L. cubeba*, and *P. americana* also experienced two WGD events^6–8^. The distribution of *K*s values in the *P. nigrum* genome showed one peak, which means that one WGD event occurred in the *P. nigrumi* genome. This is consistent with previously reported WGD results for the *P. nigrumi* genome^4^. The *K*s differentiation peak of *P. bournei*–*P. nigrum* occurred at *K*s ≈ 1.75, which was greater than the two *K*s peaks (*K*s1 ≈ 0.5–0.6 and *K*s2 ≈ 0.85–0.95) observed in Lauraceae (*P. bournei*, *C. kanehirae*, *L. cubeba*, and *P. americana*) genomes (Fig. 3a). This result indicates that after the divergence of the common ancestors of Lauraceae and Piperales (*P. nigrum*), Lauraceae experienced two WGD events. The *K*s differentiation peak of *P. bournei*–*L. chinense* (*K*s ≈ 0.825) was larger than the *K*s1 peak (*K*s1 ≈ 0.5–0.6) in Lauraceae (*P. bournei*, *C. kanehirae*, *L. cubeba*, and *P. americana*) and smaller than the *K*s2 peak (*K*s2 ≈ 0.85–0.95) in Lauraceae (*P. bournei*, *C. kanehirae*, *L. cubeba*, and *P. americana*). The results showed that an ancient WGD event (*K*s2 ≈ 0.85–0.95) occurred in Lauraceae genomes before the differentiation of *L. chinense* (Magnoliales) and Lauraceae and that a recent WGD event (*K*s1 ≈ 0.5–0.6) occurred after the differentiation of *L. chinense* (Magnoliales) and Lauraceae. The gene tree and *K*s analysis both showed that *L. chinense* experienced one WGD event and revealed two WGDs in the *P. bournei* genome (Fig. 3a; Supplementary Fig. 10). Based on the previous WGD analysis of the *L. cubeba* genome^8^, we suggest that Lauraceae has experienced two WGD events: an ancient WGD event, which may have occurred just before the divergence of Magnoliales and Lauraceae, and a recent WGD, occurring before the differentiation of Lauraceae (Fig. 3c).

### MADS-box gene family analysis

The MADS-box gene family participates in many plant processes, including floral development, flowering time determination, and fruit ripening^21^. A total of 63 MADS-box genes were identified in the *P. bournei* genome, which were classified into type I and type II MADS-box genes based on phylogenetic analysis. Thirty type I MADS-box genes were subdivided into three subfamilies: Mα, Mβ, and Mγ (Table 1; Supplementary Table 12). There were three and four members in Mγ and Mβ, respectively. The orthologs of Mα have been duplicated (23 members). Type I genes have been associated with the development of the female gametophyte, embryo^22^, and central cell and endosperm^23,24^. Their specific roles in *P. bournei* are unknown.

**Table 1:** MADS-box genes in *P. bournei*, *C. kanehirae*, and *Arabidopsis thaliana*

Category	*A. thaliana*	*P. bournei*	*C. kanehirae*
Type II (total)	33	33	37
MIKC^c^	37	27	31
MIKC^*^	6	6	6
Type I (total)	53	30	27
Mα	23	23	22
Mβ	18	4	3
Mγ	12	3	2
Total	86	63	64

**Fig. 4:**
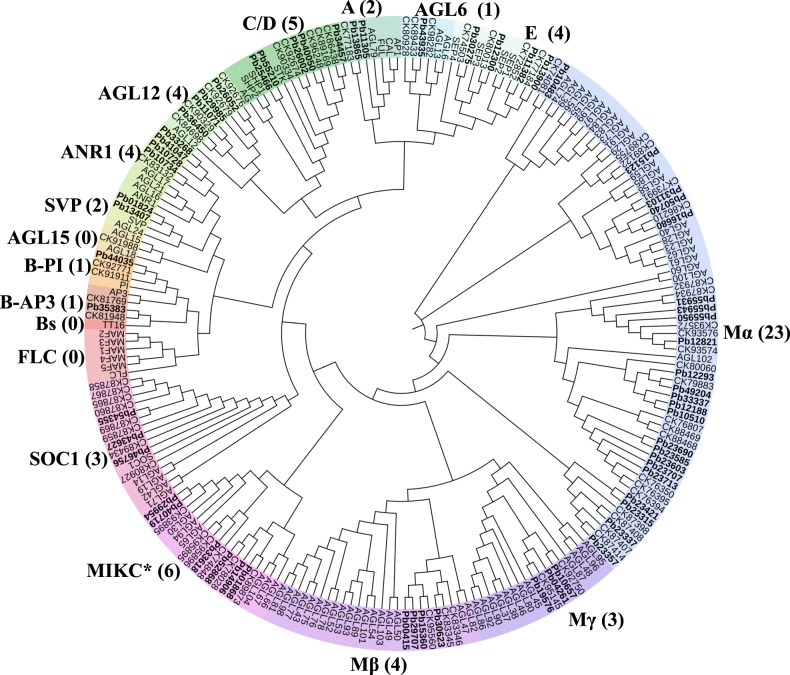
Phylogenetic analysis of MADS-box genes from *P. bournei*, *C. kanehirae*, and *A. thaliana*. The gene ID numbers begin with “Pb” to represent the gene IDs of *P. bournei*, “Ck” to represent the gene IDs of *C. kanehirae*, and “AT” to represent the gene IDs of *A. thaliana*

Type II MADS-box genes were divided into 27 MIKC^C^-type and six MIKC*-type MADS-box genes (Fig. 4; Supplementary Table 12). MIKC* regulation affects pollen gene expression^25,26^. There were fewer genes from the *SOC1*-class (three members), *A* class (two members), and *AGL6* clade (one member) than in *A. thaliana*. The *AGL12* and *ANR1* genes are involved in root development^27,28^. *P. bournei* and *A. thaliana* both contain four *ANR1* clade genes. *P. bournei* exhibits more *AGL12* genes (four members) than *A. thaliana*. *P. bournei* exhibits more genes related to root development, possibly because it requires strong roots to support its growth. However, we did not observe *FLC* subfamily genes, indicating that this family may be absent in *P. bournei*, possibly because *P. bournei* does not require vernalization for flowering, similar to rice^29^. *Bs*-class genes are usually involved in seed pigmentation and endothelium development^30^. However, there are no genes related to the *Bs* class in *P. bournei*. This difference might be related to the different fruit- and seed-development processes among species.

### GH3 and SAUR gene family analysis

**Fig. 5:**
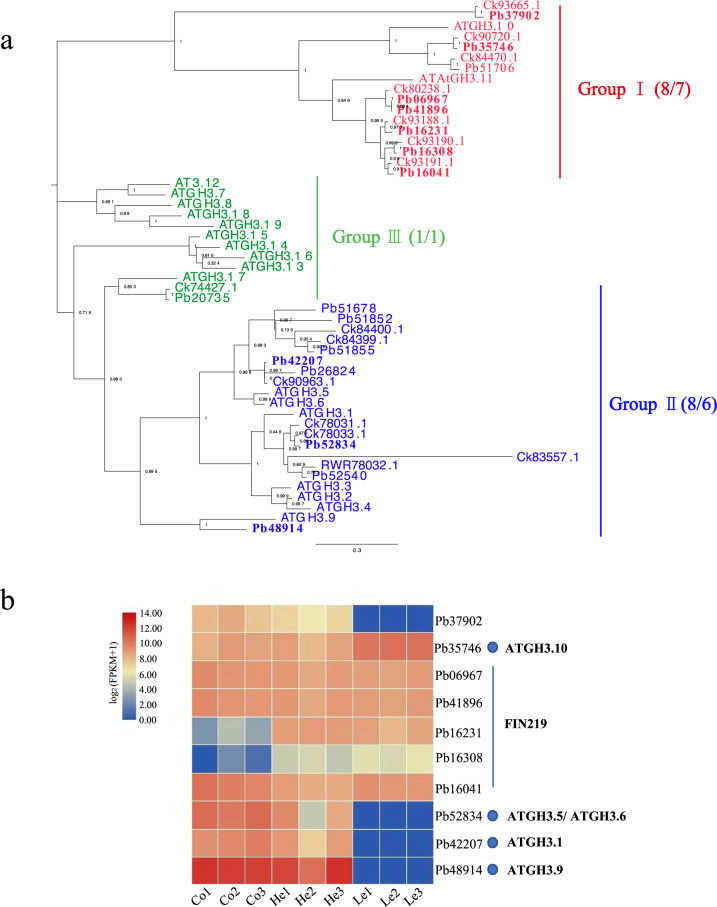
Analysis of *P. bournei* GH3 proteins. **a** Phylogenetic relationships of *P. bournei*, *C. kanehirae*, and *A. thaliana* GH3 proteins. Bootstrap values are indicated at each node. The gene ID numbers begin with “Pb“ to represent the gene IDs of *P. bournei*, “Ck” to represent the gene IDs of *C. kanehirae*, and “AT” to represent the gene IDs of *A. thaliana*. In the parentheses, the number on the left is the number of GH3 homologs in the genome of *P. bournei*, and that on the right is the number of GH3 homologs in the genome of *C. kanehirae*. **b** Expression patterns of GH3 in the cortex, heartwood, and leaf. “Co” represents the cortex, “He” represents heartwood. “Le” represents the leaf. The accession numbers of the GH3 genes of *A. thaliana* are shown in Supplementary Table 13

Auxin regulates plant growth and development by altering the expression of multiple genes^31^. Auxin-responsive genes can be divided into three classes: auxin/indoleacetic acid protein (Aux/IAA), small auxin-up RNA (SAUR), and glycoside hydrolase 3 (GH3) genes^32^. The GH3 gene family belongs to the auxin-responsive gene family. GH3 genes encode a class of acylamide synthetases that bind amino acids to indole-3-acetic acid (IAA), jasmonic acid (JA), and salicylic acid (SA). This changes the concentration of their bioactive forms in cells and regulates plant growth, development, and defense responses^33–35^. GH3 proteins are classified into three groups: group I, with JA and/or SA-amido synthetase activity; group II, with IAA–amido synthetase activity; and group III, with unknown synthetase activity^36,37^. We identified 17 and 14 GH3-class genes in *P. bournei* and *C. kanehirae*, respectively, and divided them into the three groups based on phylogenetic information (Fig. 5a). There were eight genes in groups I and II in *P. bournei*. There were seven genes in group I, and six genes in group II in *C. kanehirae*. *P. bournei* and *C. kanehirae* each exhibited one gene in group III.

*ATGH3.5*, *ATGH3.6*, and *ATGH3.1* encode IAA–amido synthetases, which help to maintain auxin homeostasis by conjugating IAA to amino acids^38^. The orthologous genes (*Pb42207* and *Pb52834*) of *ATGH3.5*, *ATGH3.6*, and *ATGH3.1* are expressed in the heartwood and cortex (Fig. 5b). We also found one *ATGH3.9*-orthologous gene, *Pb48914*, showing high expression in the heartwood and cortex (Fig. 5b). *ATGH3.9* controls auxin activity through amino acid conjugation and promotes primary root growth^39^. The *ATGH3.9*-orthologous gene in *P. bournei* shows the same function in the heartwood and cortex, thus promoting stem growth. In addition, *FIN219* (*ATGH3.11*), a phytochrome A signaling component, plays a crucial role in photomorphogenesis^40^. We identified five *FIN219*-orthologous genes in *P. bournei* and four *FIN219*-orthologous genes in *C. kanehirae* (Fig. 5a). Expression analysis showed that three *FIN219*-orthologous genes (*Pb06967*, *Pb41896*, and *Pb16041*) in *P. bournei* were expressed in the heartwood, cortex, and leaves, while one *FIN219*-orthologous (*Pb16231*) gene was expressed only in the heartwood and leaves (Fig. 5b).

**Fig. 6:**
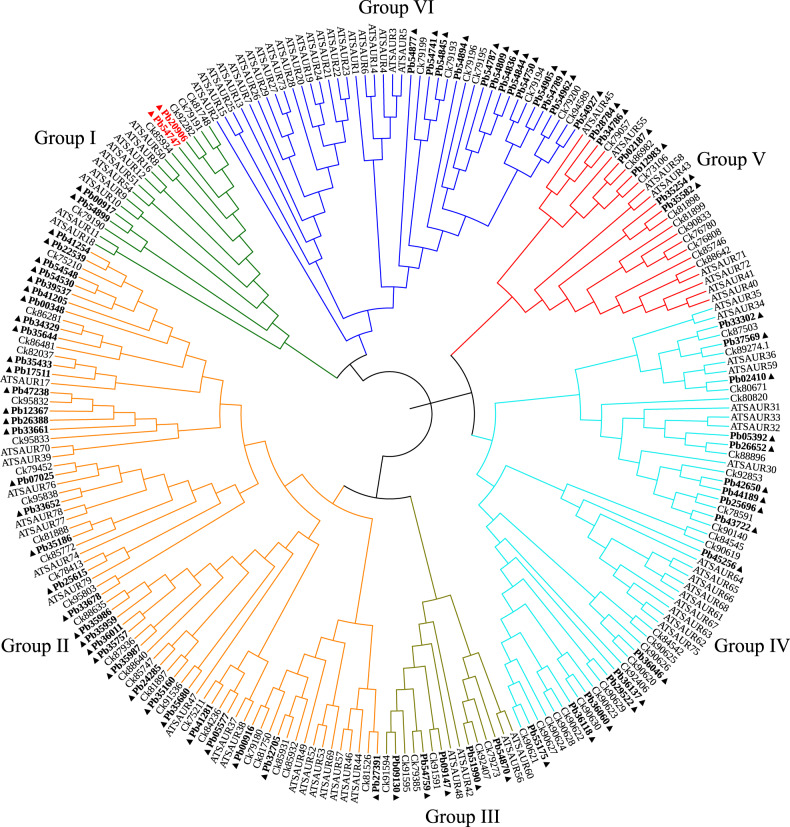
Phylogenetic relationships of *P. bournei*, *C. kanehirae*, and *A. thaliana* SAUR proteins. The gene ID numbers begin with “Pb” to represent the gene IDs of *P. bournei*, “Ck” to represent the gene IDs of *C. kanehirae*, and the “AT” to represent the gene IDs of *A. thaliana*. *SAUR50*-like genes of *P. bournei* are indicated in red

The SAUR gene family encodes highly unstable mRNA molecules with a very high turnover rate that are induced within minutes after auxin application. SAUR proteins promote cell elongation^41^. We identified 77 SAUR gene family members from *P. bournei* and 76 SAUR gene family members from *C. kanehirae* (Fig. 6). The *Arabidopsis AtSAUR50* gene is involved in light signal-mediated pedicel development^42^. In sunflower (*Helianthus annuus*), the *SAUR50*-like gene is more highly expressed on the eastern side of stems during the day, leading to sun-tracking movement during the seedling stage^43^. We also identified two *SAUR50*-like genes in *P. bournei* (*Pb20906* and *Pb54747*) and *C. kanehirae* (Fig. 6). *FIN219*-like genes and *SAUR50*-like genes in *P. bournei* may enable *P. bournei* to intercept more sunlight in dense forests, thus straightening the trunk.

### Lignin-formation analysis

**Fig. 7:**
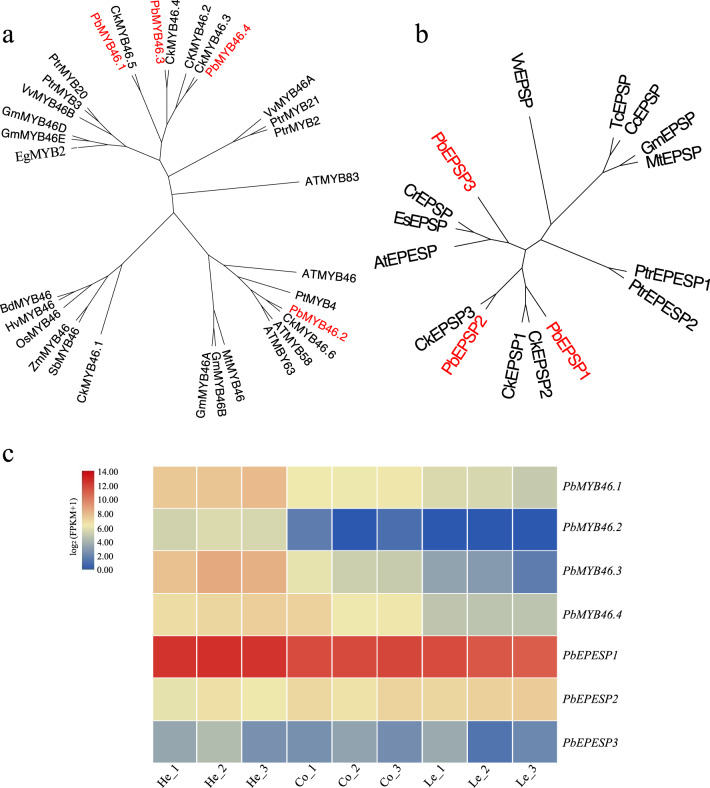
Identification of the homologous genes of *MYB46* and *EPSP*. **a** Phylogenetic relationship of *PbMYB46*-homologous genes with anther secondary wall- or lignin-associated *MYBs*. **b** Phylogenetic relationships of *PbEPSP*-homologous genes with *EPSP*-homologous genes in different plants. **c** Expression patterns of *MYB46* and *EPSP* in the cortex, heartwood, and leaves. “He” represents heartwood, “Co” represents the cortex, and “Le” represents leaves. The accession numbers of *MYB46* and *EPSP* in different species are shown in Supplementary Table 13

The principal components of wood are lignocellulosic polymers, which represent the most abundant biomass produced by terrestrial plants. Lignin biosynthesis evolved from the phenylpropanoid pathway, which promoted the successful colonization of terrestrial environments by plants^44,45^. The R2R3-MYB gene family regulates specific processes in plants, including phenylpropanoid biosynthesis^46^. *MYB46-*class genes mainly regulate the phenyl propyl pathway and lignin biosynthesis in plants. *A. thaliana MYB46* directly targets and activates the expression of multiple lignin biosynthetic genes, such as *MYB58* and *MYB63*^47^. There are four homologs of *MYB46* in *Populus trichocarpa* (*PtrMYB002*, *PtrMYB003*, *PtrMYB020*, and *PtrMYB021*), and they all activate the promoters of lignin biosynthetic genes^46,48,49^. Our phylogenetic tree showed that four *MYB46-*homologous genes and six *MYB46*-homologous genes were present in the *P. bournei* and *C. kanehirae* genomes, respectively (Fig. 7a; Supplementary Fig. 11). All *MYB46* orthologous genes from *P. bournei* were expressed in the heartwood and at low levels in the cortex, but were not in the leaves (Fig. 7c). Xie et al.^50^ found that a 5-enolpyruvylshikimate 3-phosphate synthase gene of *P. trichocarpa* (*PtrEPSP*) can directly bind to the promoter and repress the expression of a SLEEPER-like transcriptional regulator, which itself specifically binds to the promoter and represses the expression of *PtrMYB021*, thereby affecting lignin biosynthesis. We identified three *PtrEPSP*-homologous genes in the *P. bournei* and *C. kanehirae* genomes (Fig. 7b). *PbEPSP1* was highly expressed in the heartwood, cortex, and leaves, but *PbEPSP3* was not expressed (Fig. 7c).

## Conclusion

We assembled 989.19 Mb of the *P. bournei* genome and annotated 28,198 protein-coding genes. Although the genomes of four species within the magnoliid family have been sequenced, the phylogenetic relationships of the magnoliids are unclear. Our study showed that magnoliids are a sister clade to monocots and eudicots. WGD analysis suggested that Piperales and Magnoliales have both experienced one WGD event, and that Lauraceae has experienced two WGD events, where the older WGD event is shared with Magnoliales and the younger with other Lauraceae species. We identified 63 MADS-box genes in *P. bournei* and four *AGL12*-like and four *ANR1*-like genes may be related to the regulation of the roots. GH3 proteins are involved in regulating plant growth, development, and defense responses, and SAUR proteins promote cell elongation. We identified 17 members of the GH3 gene class and 77 members of the SAUR gene class in *P. bournei*. Five *FIN219*-like genes involved in photomorphogenesis and two *SAUR50*-like genes involved in light signal-mediated pedicel or stem development were identified. *MYB46* and *PtrEPSP* activate the promoters of lignin biosynthetic genes, and we identified four homologous genes of *MYB46* and three homologous genes of *PtrEPSP* in the *P. bournei* genome. These genes may be related to the formation of straight trunks in *P. bournei*. The *P. bournei* genome provides new insight into magnoliid genome evolution and diversification.

## Materials and methods

### DNA preparation and sequencing

All of the plant materials used in this study were collected from a mature *P. bournei* tree growing in Fujian Agriculture and Forestry University, Fujian Province, China. Total genomic DNA was extracted with a modified cetyltrimethylammonium bromide (CTAB) method for Illumina and de novo sequencing and assembly. Five-hundred bp paired-end libraries were constructed using the Illumina protocol. Genome size and heterozygosity were measured using GenomeScope^51^ based on a 19 *K*-mer distribution. In addition, we constructed SMRT libraries using the PacBio 20-kb protocol (https://www.pacb.com/), and they were subsequently sequenced on the PacBio platform. The transcriptomes of the heartwood, cortex, and leaves were sequenced on the Illumina platform.

### Genome assembly

Canu^52^ was used to correct errors in the original data. Flye v2.4.2^53^ was used to assemble the corrected data. Because of the high error rate of the de novo data, indel and SNP errors still existed in the assembly results. Thus, Arrow (https://github.com/PacificBiosciences/GenomicConsensus) was used to correct the assembly results. We compared the second-generation small fragment data with the assembly results, and further corrected the assembly results with Pilon v1.22^54^ to eliminate indel and SNP errors. The assembled sequence was larger than the genome size estimated through *K*-mer analysis, so we used trimDup (Rabbit Genome Assembler: https://github.com/gigascience/rabbit-genome-assembler) to remove redundancy from the assembly results. To confirm the quality of the genome assembly, we performed a BUSCO v4 (https://busco.ezlab.org)^18^ assessment using single-copy orthologous genes.

### Identification of repetitive sequences

Repetitive sequence annotation was mainly based on homologous sequence alignment and de novo assembly. Homologous sequence alignment was based on the RepBase v21.12 database^55^ (http://www.girinst.org/repbase), and RepeatMaske v4.0.7^56^ and RepeatProteinMask v4.0.7^56^ were used to identify sequences similar to known repeat sequences. We identified TEs in the *P. bournei* genome using RepeatModeler (http://www.repeatmasker.org/RepeatModeler/)^57^ and LTR_FINDER v1.06 (http://tlife.fudan.edu.cn/ltr_finder/)^58^. In addition, tandem repeats across the genome were predicted using Tandem Repeats Finder v4.09^59^ (http://tandem.bu.edu/trf/trf.html). Finally, repeat sequences with identities ≥50% were grouped into the same classes.

### Gene prediction and annotation

Two independent methods were used to predict protein-coding genes: homology-based and de novo-based prediction. Homologous proteins from nine known whole-genome sequences of *Amborella trichopoda*, *Aquilegia coerulea*, *A. thaliana*, *C. kanehirae*, *Ginkgo biloba*, *L. chinense*, *Picea abies*, *P. trichocarpa*, and *Vitis vinifera* were aligned to the *P. bournei* genome sequence using Exonerate v2.2.0 (https://www.ebi.ac.uk/Tools/psa/genewise/)^60^ for homology-based prediction. The sequences of these known genomes were downloaded from Phytozome 12 (https://phytozome.jgi.doe.gov/pz/portal.html). Two ab initio prediction software programs, Augustus^61^ (http://bioinf.uni-greifswald.de/augustus/) and SNAP^62^ (http://homepage.mac.com/iankorf), were used for de novo gene prediction. Then, the homology-based and ab initio gene structures were merged into a nonredundant gene model using Maker^63^ (http://weatherby.genetics.utah.edu/MAKER/wiki/index.php/MAKER_Tutorial_for_WGS_Assembly_and_Annotation_Winter_School_2018). We further filtered the annotated results of Maker, with the following genes filtered: (1) protein length < 50 aa and homologous protein support for exon region < 50%; and (2) CDS of the coding region and TE overlap length > 80%.

To obtain gene function information, we used BLAST v2.2.31^64^ to align the annotation results with seven protein databases, including SwissProt (http://www.uniprot.org)^65^, TrEMBL (http://www.uniprot.org/)^65^, KEGG (http://www.genome.jp/kegg/)^66^, InterPro (https://www.ebi.ac.uk/interpro/)^67^, NR, KOG^68^, and GO^69^. The tRNAs were predicted using tRNAscan-SE 1.3.1^70^. The rRNAs were identified by aligning the rRNA template sequences from the Rfam database^71^ against the genome using the BLASTN algorithm. The miRNAs and snRNAs were predicted using INFERNAL (http://infernal.janelia.org/)^72^ in Rfam, and other ncRNAs were predicted with Infernal software (http://infernal.janelia.org/)^72^ against the Rfam database.

### Genome-evolution analysis

Genes from the whole-genome sequences of 18 species (*P. bournei*, *Ananas comosus*, *A. thaliana*, *A. trichopoda*, *Phalaenopsis equestris*, *P. trichocarpa*, *Solanum lycopersicum*, *Spirodela polyrhiza*, *V. vinifera*, *L. chinense*, *Oryza sativa*, *L. cubeba*, *C. kanehirae*, *P. americana*, *Asparagus officinalis*, *Actinidia chinensis*, *Nymphaea colorata*, and *P. nigrumi*) were used for gene family-clustering analysis. We first constructed the protein data sets of these genomes and then used BLASTP (*E*-value of 1E-5) to align the protein data sets with themselves and to filter out low-quality sequences^53^. Orthologous groups present in the 18 genomes were identified using OrthoMCL v1.4 (http://orthomcl.org/orthomcl/)^73^.

MUSCLE (http://www.drive5.com/muscle/)^74^ was used to align the amino acid sequences of single-copy orthologous groups. The nucleotide sequences of the single-copy orthologous groups were connected into a supergene, and the data set was employed to construct a phylogenetic tree by using the GTR + gamma model in Mrbayes^75^. In addition, we used RAxML to combine all the data sets and constructed phylogenetic trees for protein and CDS sequences via concatenation and ASTRAL methods, respectively.

The data set employed for phylogenetic analysis was used to estimate the divergence times of each tree node using the MCMCTREE program (http://abacus.gene.ucl.ac.uk/software/paml.html) of the PAML package v4.7^76^. The nucleic acid replacement model was the GTR model, and the molecular clock model was the independent rate model. The MCMC process consisted of 500,000 burn-in iterations and 500,0000 sampling iterations (sampling every 100 iterations). The same parameters were executed twice to obtain a more stable result. Published data from *Lemna minor*–*O. sativa* (117–140 Mya), magnolias (112.6 Mya), monocots–dicots (140 Mya), and angiosperms (200 Mya) were used to calibrate divergence times^77,78^.

We used CAFÉ 4.2 software (http://sourceforge.net/projects/cafehahnlab/)^79^ to measure the expansion and contraction of orthologous gene families. Based on the maximum-likelihood modeling of gene gains and losses, we analyzed gene families for signs of expansion or contraction using genome data from 18 species.

### Collinearity analysis and whole-genome duplication

Within collinear segments, genes are conserved in function and sequence, and these genes remain highly conserved during the evolution of species. We used the default parameters of JCVI v0.9.14 (https://pypi.org/project/jcvi/)^80^ to analyze the protein sequences of *P. bournei*, *P. americana*, *C. kanehirae*, *L. cubeba*, *L. chinense*, and *P. nigrum*, and obtained the gene pairs in the collinear regions. We used *K*s distribution analysis to estimate WGD events in the *P. bournei*, *P. americana*, *C. kanehirae*, *L. cubeba*, *L. chinense*, and *P. nigrum* genomes. Diamond was used to conduct self-alignment of the protein sequences of these species genomes and then to extract the mutual optimal alignment in the alignment results. Finally, Codeml in the PAML package was used to calculate *K*s values^81,82^.

Our *K*s analysis showed that the genomes of *P. bournei* presented two *K*s peaks (*K*s1 ≈ 0.5–0.6 and *K*s2 ≈ 0.85–0.95), whereas that of *L. chinense* only exhibited one (*K*s ≈ 0.7), and *P. bournei*–*L. chinense* showed one differentiation peak (*K*s ≈ 0.825) (Fig. 3). Therefore, to determine the differentiation of *P. bournei* and *L. chinense*, we constructed a gene tree. For the construction of the gene tree, Blastp (e-value < 1e-5)^53^ was first used to align the protein sequences of all pairs of genes in the *P. bournei* genome equal to the *K*s peak of *P. bournei* itself with those in the *L. chinense* genome equal to the *K*s peak of *L. chinense* itself. Then, according to the alignment result, the genes meeting one of the following two conditions were selected: gene pairs of the *K*s2 peak in the *P. bournei* genome were aligned to the gene pairs of the *K*s peak in the *L. chinense* genome; the gene pairs of the *K*s1 and *K*s2 peaks in the *P. bournei* genome were aligned to the gene pairs of the *K*s peak in the *L. chinense* genome. We selected nine gene pairs that satisfied the first condition and two gene pairs that satisfied the second condition. Finally, the selected gene pairs were employed to construct a gene tree using RaxMLv8 (parameter -m PROTGAMMAJTT), and the outgroup was fixed as evm_27.model.amtr_v1.0_scaffold00106.118 of *A. trichopoda*^83^.

### Gene family analysis

The candidate sequences of the MADS-box and SAUR genes of *A. thaliana* were downloaded from TAIR (https://www.arabidopsis.org/index.jsp). The HMM profiles of the MADS (PF00319)^84^, GH3 auxin-responsive promoter (PF03321)^85^, and SAUR (PF02519)^86^ gene families were obtained from Pfam (http://pfam.xfam.org/). Each protein of the gene families of *P. bournei* and *C. kanehirae* was separately searched with the HMMER 3.2.1 (with default parameters)^87^ and BLASTP (*E*-value of e^−5^)^53^ methods in the *P. bournei* and *C. kanehirae* genomes. The whole-genome sequence of *C. kanehirae* was extracted from NCBI (Bioproject: PRJNA477266). The protein sequence set for the MADS-box gene candidates of *P. bournei* was employed for BLAST analysis against the assembled *P. bournei* transcriptomes with the TBLASTN program^53^. Subsequently, all of the candidate sequences of the MADS-box, GH3, and SAUR genes were subjected to SMART analysis (http://smart.embl-heidelberg.de/)^88^. The candidate MADS-box, GH3, and SAUR gene families were aligned with MEGA5^89^, and the phylogenetic tree was constructed on the CIPRES website (https://www.phylo.org/portal2/). The MADS-box, CH3, and SAUR phylogenetic trees were visualized using iTOL (https://itol.embl.de).

## Supplementary information

The Phoebe genome sheds light on the evolution of the magnoliids

## Data Availability

Genome sequences have been submitted to the National Genomics Data Center (NGDC). The raw whole-genome data of *P. bournei* have been deposited in BioProject/GSA (https://bigd.big.ac.cn/gsa.)^90^ under the accession codes PRJCA002001/CRA002192, and the assembly and annotation of the whole-genome data have been deposited at BioProject/GWH (https://bigd.big.ac.cn/gwh)^91^ under the accession codes PRJCA002001/GWHACDM00000000.
