# Supplementary material for: The Phoebe genome sheds light on the evolution of magnoliids
Source: Hortic Res. 2020 Sep 1;7:146. doi: 10.1038/s41438-020-00368-z (PMC7459323; doi:10.1038/s41438-020-00368-z)
Supplement: Supplementary file 1 — The Phoebe genome sheds light on the evolution of the magnoliids [file 41438_2020_368_MOESM1_ESM.docx]

**Supplementary Information**

**The *Phoebe* genome sheds light on the evolution of the magnoliids**

Shi-Pin Chen^1,2^, Wei-Hong Sun^1,2^, Yuan-Fang Xiong^1,2^, Yu-Ting Jiang^1,2^, Xue-Die Liu^1,2^, Xing-Yu Liao^1,2^, Di-Yang Zhang^1,^^2^, Shu-Zhen Jiang^1,2^, Yu Li^1,2^, Bin Liu^1,2^, Liang Ma^2^, Xia Yu^2^, Li He^2^, Bao Liu^2^, Jin-Lin Feng^2^, Li-Zhen Feng^2^, Zhiwen Wang^3^, Shung-Quan Zou^1,2^, Si-Ren Lan^1,2^, and Zhong-Jian Liu^2†^

Running title: Nanmu genome

**Content**

[Supplementary Figures 3](#_Toc42174006)

[Supplementary Figure 1. Genome size and heterozygosity estimation using 19 K-mer distribution. 3](#_Toc42174007)

[Supplementary Figure 2. Read length distribution of PacBio sequencing data. 4](#_Toc42174008)

[Supplementary Figure 3. Gene structure prediction results statistics. 5](#_Toc42174009)

[Supplementary Figure 4. The sequence divergence rate of four different TEs using RepeatMasker annotation. 6](#_Toc42174010)

[Supplementary Figure 5. The sequence divergence rate of four different TEs using *de novo* annotation. 7](#_Toc42174011)

[Supplementary Figure 6. The insert time of LTR. 8](#_Toc42174012)

[Supplementary Figure 7. Comparison of number of homologous genes between the genomes of 18 species. 9](#_Toc42174013)

[Supplementary Figure 8. Comparison of q-value of phylogenetic trees based on astral using nucleotides and amino acids. 10](#_Toc42174015)

[Supplementary Figure 9. Divergence times between *P. bournei* and other plant species. 11](#_Toc42174017)

[Supplementary Figure 10. The gene tree of *P. bournei* and *L. chinense*. 12](#_Toc42174019)

[Supplementary Figure 11. Phylogenetic analysis MYB genes from *P. bournei*, *C. kanehirae*, and *A. thaliana.* 13](#_Toc42174022)

[Supplementary Tables 14](#_Toc42174024)

[Supplementary Table 1. The statistics of sequencing raw data from Illumina sequencing. 14](#_Toc42174025)

[Supplementary Table 2. The statistics of sequencing raw data from Pacific Biosciences platforms. 15](#_Toc42174026)

[Supplementary Table 3. Assembly statistics of the *P. bournei*, *C. kanehirae*, and *P. americana* genomes. 16](#_Toc42174027)

[Supplementary Table 4. Illumina sequence alignment statistics. 17](#_Toc42174028)

[Supplementary Table 5. The prediction of gene structures of the *P. bournei*. 18](#_Toc42174029)

[Supplementary Table 6. BUSCO assessment of the *P. bournei* genome 19](#_Toc42174030)

[Supplementary Table 7. Statistics on the annotation of non-coding RNA of the *P. bournei* genome. 20](#_Toc42174031)

[Supplementary Table 8. The statistic result of repeat sequence. 21](#_Toc42174032)

[Supplementary Table 9. Statistic of repeat sequence in *P. bournei*. 22](#_Toc42174033)

[Supplementary Table 10. The statistics results of function annotation. 23](#_Toc42174034)

[Supplementary Table 11. Statistic result of clustered gene families. 24](#_Toc42174035)

[Supplementary Table 12. List of MADS-box genes identified in *P. bournei* and *C. micranthum*. 25](#_Toc42174036)

[Supplementary Table 13. List of reference GH3, MYB46, and EPSP genes from different species. 27](#_Toc42174037)

# Supplementary Figures

## Supplementary Figure 1. Genome size and heterozygosity estimation using 19 K-mer distribution.

## Supplementary Figure 2. Read length distribution of PacBio sequencing data.

## Supplementary Figure 3. Gene structure prediction results statistics.

*P. bournei* compared with genetic elements of related species. Window refers to the length represented by each point on the horizonal coordinate.

## Supplementary Figure 4. The sequence divergence rate of four different TEs using RepeatMasker annotation.

## Supplementary Figure 5. The sequence divergence rate of four different TEs using *de novo* annotation.

## Supplementary Figure 6. The insert time of LTR.

The horizontal axis represents the time of LTR insertion, and the vertical axis represents the number of LTR, Copia, and Gypsy.

**
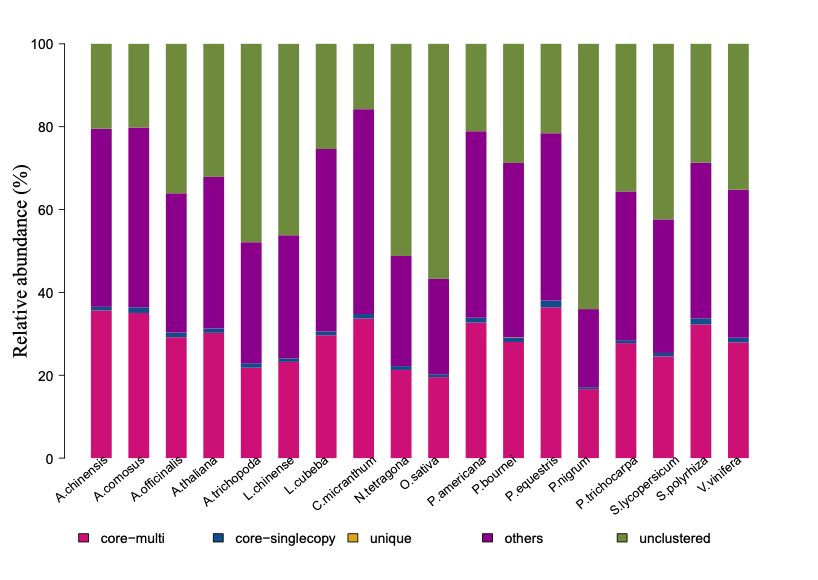
**

## Supplementary Figure 7. Comparison of number of homologous genes between the genomes of 18 species.

##
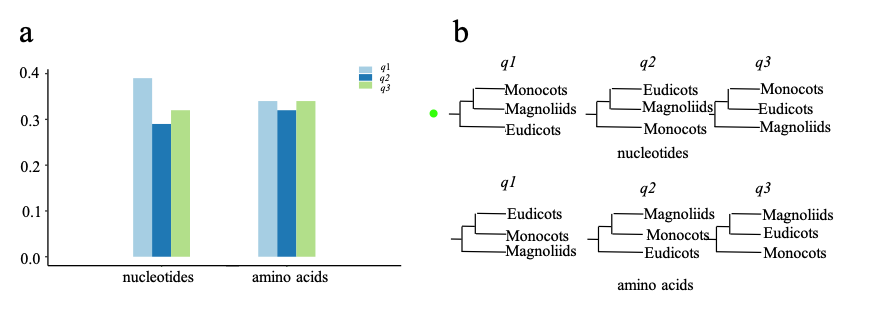


## Supplementary Figure 8. Comparison of q-value of phylogenetic trees based on astral using nucleotides and amino acids.

## a. The q-values of the two branches in phylogenetic based on astral nucleotides and amino acids. From a, we found there is little difference between q1, q2, and q3. b. The branch structure of binary tree based on astral using nucleotides and amino acids under three possibilities of q1, q2 and q3.


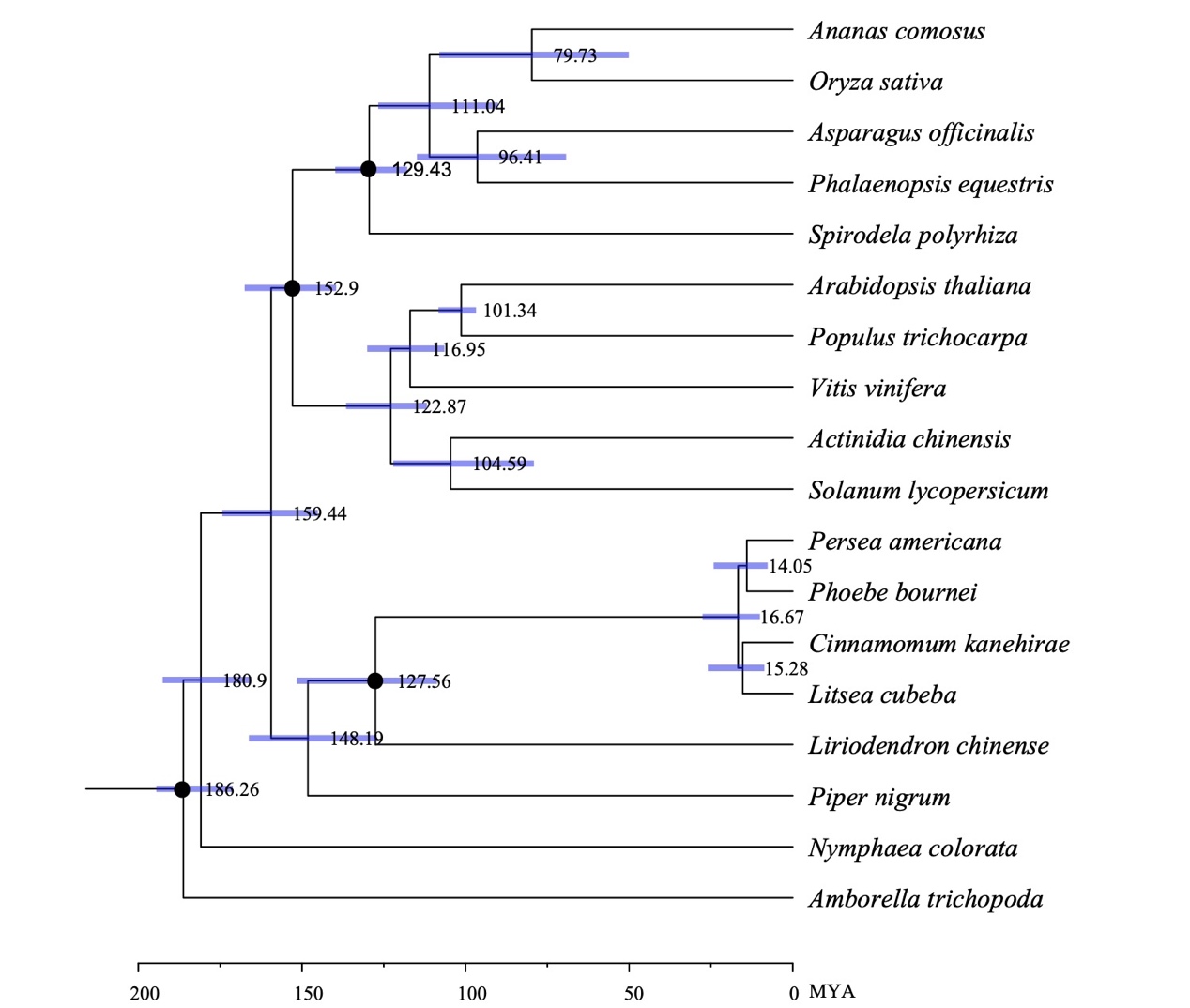


## Supplementary Figure 9. Divergence times between *P. bournei* and other plant species.

##

## Supplementary Figure 10. The gene tree of *P. bournei* and *L. chinense*.

## The gene ID number begins with ‘ATR’ to represent the gene ID of *Amborelle*; the gene ID number begins with ‘LCHIIL’ to represent the gene ID of *L. cubeba*, and the gene ID number begins with ‘PBOUI’ to represent the gene ID of *P. bournei*.

##

## Supplementary Figure 11. Phylogenetic analysis MYB genes from *P. bournei*, *C. kanehirae*, and *A. thaliana.*

## The red branch is a homologous gene of *MYB46*. The purple marker gene ID is a homologous gene of MYB46 in *P. bournei*; and the orange marker gene ID is a homologous gene of MYB46 in *C. kanehirae*.

# Supplementary Tables

## Supplementary Table 1. The statistics of sequencing raw data from Illumina sequencing.

| **Lib ID** | **Insert Size (bp)** | **Read Length (bp)** | **Raw Data (Gb)** | **Clean Data (Gb)** |
| --- | --- | --- | --- | --- |
| Whaipi106555-35 | 500 | 150 | 102.05 | 84.23 |

## Supplementary Table 2. The statistics of sequencing raw data from Pacific Biosciences platforms.

| **ID** | **ZMWNUM** | **Total bases (Gb)** | **Total reads** | **Average length (bp)** | **Max length (bp)** | **N50** |
| --- | --- | --- | --- | --- | --- | --- |
|  |  |  |  |  |  | **length** |
|  |  |  |  |  |  | **(bp)** |
| r54040_20190717_100522-1_A01 | 708,899 | 17.19 | 1,417,799 | 12127.78 | 191,149 | 18,419 |
| r54040_20190719_085139-1_A01 | 680,528 | 16.8 | 1,398,432 | 12010.6 | 225,480 | 18,464 |
| r54040_20190725_075333-1_A01 | 618,658 | 14.82 | 1,199,289 | 12357.74 | 215,655 | 18,533 |
| r54266_20190720_083212-1_C01 | 622,315 | 15.11 | 1,205,488 | 12533.11 | 197,055 | 18,542 |
| r54267_20190720_083121-1_D01 | 639,901 | 15.52 | 1,252,841 | 12390.43 | 238,687 | 18,530 |
| r54268_20190723_034946-1_B01 | 648,630 | 14.03 | 1,136,677 | 12344.13 | 203,732 | 18,434 |
| r54270_20190723_034850-1_A01 | 694,155 | 16.36 | 1,327,616 | 12321.06 | 249,553 | 18,425 |
| total | - | 109.83 | 8,938,142 | 12288.01 | 249,553 | 18,477 |

## Supplementary Table 3. Assembly statistics of the *P. bournei*, *C. kanehirae*, and *P. americana* genomes.

| **Metrics** | ***P. bournei*** | ***C. kanehirae*** | ***P. americana*** | |
| --- | --- | --- | --- | --- |
|  |  |  | **Var. *drymifolia*** | **Hass cultivar** |
| **Contig N50** | 2,053,022 bp | 0.9 Mb | 11,724 bp | 296,371 bp |
| **Longest Contig** | 10,155,688 bp | - | 254,240 bp | 2,811,280 bp |
| **Total Contig length** | 989,189,349 bp | - | 668,137,248 bp | 912,697,600 bp |
| **N50 Scaffold** | 2,053,022 bp | 50.4 Mb | 323,854 bp | - |
| **Longest Scaffold** | 10,155,688 bp | - | 4,610,966 bp | - |
| **Total Scaffold length** | 989,189,349 bp | - | 823,419,498 bp | - |
| **BUSCO** | 95.0% | 88.5% | 86.3% | 85% |

The assembly data of the *C. kanehirae* and *P. americana* genomes are respectively frome the research reported of Shu et al and Martha et al.

## Supplementary Table 4. Illumina sequence alignment statistics.

|  | **Number** | **Ratio (%)** |
| --- | --- | --- |
| **Total Reads** | 566,612,752 | - |
| **Mapped Reads** | 560,199,891 | 98.87 |
| **Mapped and Paired Reads** | 559,680,494 | 98.78 |

**Total reads:** reads statistics of filtered data;

**Mapped Reads：**Reads statistics of matched genomes;

**Mapped and Paired Reads:** Reads statistics of mapped genomes and paired.

## Supplementary Table 5. The prediction of gene structures of the *P. bournei*.

|  | **Gene set** | **Number** | **Average mRNA length(bp)** | **Average CDS length(bp)** | **Average**  **exon per gene** | **Average exon length(bp)** | **Average intron length (bp)** |
| --- | --- | --- | --- | --- | --- | --- | --- |
| **Denovo** | **Augustus** | 37,254 | 8346.77 | 1139.31 | 4.93 | 231.29 | 1835.84 |
|  | **SNAP** | 67,248 | 13272.48 | 950.09 | 5.85 | 162.47 | 2541.84 |
| **Homolog** | ***A. trichopoda*** | 66,468 | 4104.80 | 771.18 | 2.96 | 260.27 | 1698.19 |
|  | ***A. coerulea*** | 80,593 | 5206.70 | 968.47 | 3.37 | 286.98 | 1784.80 |
|  | ***A. thaliana*** | 65,595 | 5254.88 | 937.00 | 3.64 | 257.30 | 1634.58 |
|  | ***C. kanehirae*** | 120,997 | 5519.48 | 1028.2 | 3.49 | 294.47 | 1802.33 |
|  | ***G. biloba*** | 126,420 | 4060.44 | 1307.28 | 2.39 | 547.58 | 1984.460 |
|  | ***L. chinense*** | 102,106 | 5157.74 | 965.89 | 3.24 | 297.90 | 1869.45 |
|  | ***P. abies*** | 196,262 | 2431.90 | 654.64 | 1.96 | 334.36 | 1855.37 |
|  | ***P. trichocarpa*** | 123,619 | 4863.47 | 897.90 | 3.28 | 273.52 | 1737.22 |
|  | ***V. vinifera*** | 105,763 | 4542.34 | 849.14 | 3.03 | 280.57 | 1822.47 |
| **Maker** | **-** | 28,198 | 9695.55 | 1258.80 | 5.59 | 225.24 | 1836.58 |

## Supplementary Table 6. BUSCO assessment of the *P. bournei* genome

| **Type** | **Number** | **Percentage** |
| --- | --- | --- |
| **Complete BUSCOs（C）** | 1,115 | 81.1% |
| **Complete and single-copy BUSCOs (S)** | 1,040 | 75.7% |
| **Complete and duplicated BUSCOs (D)** | 74 | 5.4% |
| **Fragmented BUSCOs (F)** | 89 | 6.5% |
| **Missing BUSCOs (M)** | 171 | 12.4% |
| **Total BUSCO groups searched** | 1,375 | - |

## Supplementary Table 7. Statistics on the annotation of non-coding RNA of the *P. bournei* genome.

| **Type** | | **Copy** | **Average length (bp)** | **Total length (bp)** | **% of genome** |
| --- | --- | --- | --- | --- | --- |
| **miRNA** | | 145 | 124.482759 | 18,050 | 0.001825 |
| **tRNA** | | 813 | 75.065191 | 61,028 | 0.006169 |
| **rRNA** | **rRNA** | 2,417 | 160.786926 | 388,622 | 0.039287 |
|  | **18S** | 145 | 1286.468966 | 186,538 | 0.018858 |
|  | **28S** | 421 | 140.192400 | 59,021 | 0.005967 |
|  | **5.8S** | 105 | 151.580952 | 15,916 | 0.001609 |
|  | **5S** | 1,746 | 72.821879 | 127,147 | 0.012854 |
|  | **snRNA** | 519 | 120.136802 | 62,351 | 0.006303 |
|  | **CD-box** | 294 | 101.843537 | 29,942 | 0.003027 |
| **snRNA** | **HACA-box** | 32 | 125.281250 | 4,009 | 0.000405 |
|  | **splicing** | 193 | 147.150260 | 28,400 | 0.002871 |

## Supplementary Table 8. The statistic result of repeat sequence.

| **Type** | **Repeat Size (bp)** | **% of genome** |
| --- | --- | --- |
| **TRF** | 63,831,547 | 6.45 |
| **RepeatMasker** | 113,416,607 | 11.47 |
| **RepeatProteinMask** | 135,994,612 | 13.75 |
| **De novo** | 664,020,101 | 67.13 |
| **Total** | 677,685,434 | 68.51 |

## Supplementary Table 9. Statistic of repeat sequence in *P. bournei*.

|  | **RepBase Tes** | | **TE Proteins** | | **De novo** | | **Combined Tes** | |
| --- | --- | --- | --- | --- | --- | --- | --- | --- |
|  | **Length** | **%in** | **Length** | **% in** | **Length** | **% in** | **Length** | **% in** |
|  | **(bp)** | **Genome** | **(bp)** | **Genome** | **(bp)** | **Genome** | **(bp)** | **Genome** |
| **DNA** | 18,330,883 | 1.853122 | 18,431,196 | 1.863263 | 46,847,332 | 4.735932 | 61,049,880 | 6.171708 |
| **LINE** | 12,696,301 | 1.283506 | 18,366,643 | 1.856737 | 54,181,140 | 5.477327 | 60,371,080 | 6.103086 |
| **LINE/L1** | 6,129,046 | 0.619603 | 14,528,674 | 1.468745 | 38,080,969 | 3.849715 | 42,429,940 | 4.289365 |
| **LINE/RTE** | 5,538,365 | 0.559889 | 3,807,858 | 0.384947 | 13,140,365 | 1.328397 | 14,167,402 | 1.432223 |
| **SINE** | 215,173 | 0.021752 | 0 | 0 | 0 | 0 | 215,173 | 0.021752 |
| **LTR** | 83,917,981 | 8.483510 | 99,887,723 | 10.097938 | 514,520,341 | 52.014343 | 518,967,594 | 52.463929 |
| **LTR/Copia** | 35,974,648 | 3.636781 | 36,844,102 | 3.724676 | 129,525,422 | 13.094098 | 135,154,708 | 13.663179 |
| **LTR/Gypsy** | 46,038,869 | 4.654202 | 62,593,307 | 6.327738 | 245,144,462 | 24.782360 | 249,454,508 | 25.218075 |
| **Other** | 1,111 | 0.000112 | 0 | 0 | 0 | 0 | 1,111 | 0.000112 |
| **Unknown** | 0 | 0 | 0 | 0 | 83,645,491 | 8.455964 | 83,645,491 | 8.455964 |
| **Total** | 113,416,607 | 11.465611 | 135,994,612 | 13.748087 | 645,042,802 | 65.209234 | 651,643,082 | 65.876476 |

*LINE, long interspersed nuclear element; SINE, short interspersed element; LTR, long terminal repeat;

*Denovo+Repbase denotes transposable elements identified by RepeatMasker (<http://www.repeatmasker.org>) with default options after RepeatModeler/RepeatScout/Piler/LTR_finder software use with RepBase database prediction.

*TE proteins were transposable elements identified in the genome through the annotation of Repeat ProteinMask software using the RepBase database.

*Combined TEs involved a combination of the above two methods.

*Unknown repeat sequences could not be clustered by Repeat Masker.

## Supplementary Table 10. The statistics results of function annotation.

| **Values** | **Total** | **Nr** | **Swissprot** | **KEGG** | **KOG** | **TrEMBL** | **Interpro** | **GO** | **Overall** |
| --- | --- | --- | --- | --- | --- | --- | --- | --- | --- |
| **Number** | 28,198 | 25,111 | 20,453 | 19,649 | 19,417 | 26,924 | 24,080 | 15,470 | 27,011 |
| **Percentage** | **-** | 89.05% | 72.53% | 69.68% | 68.86% | 95.48% | 85.40% | 54.86% | 95.79% |

## Supplementary Table 11. Statistic result of clustered gene families.

| **Species** | **Genes** | **Unclustered genes** | **Clustered genes** | **Families** | **Unique families** | **Unique families**  **genes** | **Common families** | **Common**  **families**  **genes** | **Single copy** | **Average genes per family** |
| --- | --- | --- | --- | --- | --- | --- | --- | --- | --- | --- |
| ***A.chinensis*** | 32962 | 6753 | 26209 | 13527 | 0 | 0 | 4678 | 12036 | 292 | 1.938 |
| ***A.comosus*** | 21445 | 4331 | 17114 | 12109 | 0 | 0 | 4678 | 7806 | 292 | 1.413 |
| ***A.officinalis*** | 26005 | 9381 | 16624 | 11732 | 0 | 0 | 4678 | 7882 | 292 | 1.417 |
| ***A.thaliana*** | 27404 | 8802 | 18602 | 12407 | 0 | 0 | 4678 | 8578 | 292 | 1.499 |
| ***A.trichopoda*** | 26846 | 12858 | 13988 | 11955 | 0 | 0 | 4678 | 6149 | 292 | 1.17 |
| ***L.chinense*** | 35269 | 16298 | 18971 | 12569 | 0 | 0 | 4678 | 8484 | 292 | 1.509 |
| ***L.cubeba*** | 31327 | 7954 | 23373 | 14914 | 0 | 0 | 4678 | 9583 | 292 | 1.567 |
| ***C.micranthum*** | 26531 | 4188 | 22343 | 14241 | 0 | 0 | 4678 | 9227 | 292 | 1.569 |
| ***N.tetragona*** | 31589 | 16175 | 15414 | 11632 | 0 | 0 | 4678 | 7014 | 292 | 1.325 |
| ***O.sativa*** | 42189 | 23932 | 18257 | 12207 | 0 | 0 | 4678 | 8538 | 292 | 1.496 |
| ***P.americana*** | 24616 | 5195 | 19421 | 13657 | 0 | 0 | 4678 | 8344 | 292 | 1.422 |
| ***P.bournei*** | 28198 | 8109 | 20089 | 13883 | 1 | 21 | 4678 | 8196 | 292 | 1.447 |
| ***P.equestris*** | 17870 | 3858 | 14012 | 10795 | 0 | 0 | 4678 | 6797 | 292 | 1.298 |
| ***P.nigrum*** | 63466 | 40638 | 22828 | 12245 | 0 | 0 | 4678 | 10863 | 292 | 1.864 |
| ***P.trichocarpa*** | 41331 | 14727 | 26604 | 13873 | 0 | 0 | 4678 | 11767 | 292 | 1.918 |
| ***S.lycopersicum*** | 34682 | 14696 | 19986 | 13300 | 0 | 0 | 4678 | 8802 | 292 | 1.503 |
| ***S.polyrhiza*** | 19591 | 5623 | 13968 | 11222 | 0 | 0 | 4678 | 6610 | 292 | 1.245 |
| ***V.vinifera*** | 26346 | 9280 | 17066 | 12490 | 0 | 0 | 4678 | 7656 | 292 | 1.366 |

Unclustered genes refer to the number of genes endemic to the species. Unique families refer to the gene family that are Unique to a species.

## Supplementary Table 12. List of MADS-box genes identified in *P. bournei*.

| Gene ID | Name | Type | Subfamily | ORF (pb) | length | MV(Da) | pI |
| --- | --- | --- | --- | --- | --- | --- | --- |
| Maker00013865 | Pb13865 | MIKCc | A | 254 | 211 | 24187.3 | 10.5 |
| Maker00011305 | Pb11305 | MIKCc | A | 188 | 147 | 17261.7 | 9.98 |
| Maker00043935 | Pb43935 | MIKCc | AGL6 | 439 | 394 | 44552.6 | 8.43 |
| Maker00036450 | Pb36450 | MIKCc | AGL12 | 178 | 175 | 20114.5 | 8.84 |
| Maker00011071 | Pb11071 | MIKCc | AGL12 | 126 | 122 | 14204.7 | 10.39 |
| Maker00026052 | Pb26052 | MIKCc | AGL12 | 282 | 249 | 28433.8 | 7.46 |
| Maker00029985 | Pb29985 | MIKCc | AGL12 | 130 | 96 | 10968.6 | 10.72 |
| Maker00010728 | Pb10728 | MIKCc | ANR1 | 255 | 186 | 19635.9 | 8.66 |
| Maker00010734 | Pb10734 | MIKCc | ANR1 | 182 | 142 | 16550.9 | 9.86 |
| Maker00041047 | Pb41047 | MIKCc | ANR1 | 205 | 174 | 20128.7 | 9.95 |
| Maker00033268 | Pb33268 | MIKCc | ANR1 | 158 | 118 | 13009.2 | 8.77 |
| Maker00044035 | Pb44035 | MIKCc | B-P1 | 322 | 249 | 28878.1 | 9.03 |
| Maker00035383 | Pb35383 | MIKCc | B-AP3 | 256 | 217 | 25215.6 | 9.42 |
| Maker00055210 | Pb55210 | MIKCc | C/D | 236 | 192 | 22001.6 | 9.92 |
| Maker00040050 | Pb40050 | MIKCc | C/D | 202 | 156 | 17945.5 | 10.4 |
| Maker00034457 | Pb34457 | MIKCc | C/D | 275 | 240 | 27386.5 | 9.73 |
| Maker00026002 | Pb26002 | MIKCc | C/D | 270 | 223 | 25550.9 | 9.76 |
| Maker00025466 | Pb25466 | MIKCc | C/D | 287 | 236 | 27198.3 | 10.25 |
| Maker00012300 | Pb12300 | MIKCc | E | 118 | 88 | 10138.6 | 10.24 |
| Maker00011382 | Pb11382 | MIKCc | E | 288 | 258 | 30070.3 | 9.19 |
| Maker00013900 | Pb13900 | MIKCc | E | 251 | 212 | 23780.7 | 4.67 |
| Maker00030275 | Pb30275 | MIKCc | E | 289 | 237 | 27189.7 | 9.31 |
| Maker00043627 | Pb43627 | MIKCc | SOC1 | 210 | 173 | 19971.4 | 10.8 |
| Maker00054355 | Pb54355 | MIKCc | SOC1 | 321 | 255 | 29234.4 | 10.4 |
| Maker00046756 | Pb46756 | MIKCc | SOC1 | 156 | 132 | 15443.1 | 10.6 |
| Maker00001824 | Pb01824 | MIKCc | SVP | 304 | 266 | 30319.6 | 9.44 |
| Maker00013407 | Pb13407 | MIKCc | SVP | 236 | 206 | 23388.3 | 5.22 |
| Maker00052868 | Pb52868 | MIKC* |  | 339 | 297 | 33777.2 | 8.77 |
| Maker00033618 | Pb33618 | MIKC* |  | 378 | 291 | 33027 | 9.71 |
| Maker00001868 | Pb01868 | MIKC* |  | 376 | 308 | 35124.2 | 5.74 |
| Maker00014906 | Pb14906 | MIKC* |  | 154 | 138 | 16191.6 | 9.07 |
| Maker00029954 | Pb29954 | MIKC* |  | 666 | 538 | 61276.5 | 6.65 |
| Maker00040719 | Pb40719 | MIKC* |  | 523 | 388 | 44439.3 | 8.72 |
| Maker00023315 | Pb23315 | Type I | Mα | 426 | 333 | 37462.9 | 6.63 |
| Maker00023337 | Pb23337 | Type I | Mα | 426 | 343 | 39000 | 4.31 |
| Maker00023357 | Pb23357 | Type I | Mα | 350 | 272 | 30142.8 | 4.34 |
| Maker00023421 | Pb23421 | Type I | Mα | 730 | 575 | 64249.7 | 4.34 |
| Maker00031103 | Pb31103 | Type I | Mα | 281 | 224 | 24959.7 | 7.88 |
| Maker00015123 | Pb15123 | Type I | Mα | 210 | 171 | 19097.5 | 4.79 |
| Maker00023585 | Pb23585 | Type I | Mα | 998 | 769 | 83697.4 | 4.16 |
| Maker00023603 | Pb23603 | Type I | Mα | 472 | 366 | 39610 | 4.05 |
| Maker00023690 | Pb23690 | Type I | Mα | 1029 | 799 | 86796.6 | 4.31 |
| Maker00023707 | Pb23707 | Type I | Mα | 394 | 310 | 33739.8 | 4.14 |
| Maker00023713 | Pb23713 | Type I | Mα | 564 | 433 | 47029.3 | 4.28 |
| Maker00010483 | Pb10483 | Type I | Mα | 322 | 245 | 26794.7 | 5.95 |
| Maker00010510 | Pb10510 | Type I | Mα | 94 | 78 | 8845 | 9.2 |
| Maker00016680 | Pb16680 | Type I | Mα | 275 | 217 | 24426 | 10.16 |
| Maker00049204 | Pb49204 | Type I | Mα | 712 | 535 | 59687.2 | 8.7 |
| Maker00012821 | Pb12821 | Type I | Mα | 465 | 352 | 39339 | 8.31 |
| Maker00055931 | Pb55931 | Type I | Mα | 552 | 413 | 46847.5 | 6.74 |
| Maker00055943 | Pb55943 | Type I | Mα | 496 | 372 | 41370.5 | 4.21 |
| Maker00055950 | Pb55950 | Type I | Mα | 502 | 376 | 42003.2 | 4.43 |
| Maker00050740 | Pb50740 | Type I | Mα | 274 | 217 | 24364 | 10.29 |
| Maker00033337 | Pb33337 | Type I | Mα | 377 | 295 | 32237.2 | 7.43 |
| Maker00012188 | Pb12188 | Type I | Mα | 174 | 157 | 17925.8 | 4.36 |
| Maker00012293 | Pb12293 | Type I | Mα | 202 | 166 | 18992.7 | 10.32 |
| Maker00015360 | Pb15360 | Type I | Mβ | 394 | 342 | 39617 | 5.71 |
| Maker00030623 | Pb30623 | Type I | Mβ | 846 | 661 | 76107.9 | 8.79 |
| Maker00029707 | Pb29707 | Type I | Mβ | 672 | 555 | 61891.9 | 4.97 |
| Maker00000415 | Pb00415 | Type I | Mβ | 787 | 652 | 72881.2 | 4.81 |
| Maker00004261 | Pb04261 | Type I | Mγ | 459 | 351 | 40132.8 | 6.43 |
| Maker00019576 | Pb19576 | Type I | Mγ | 241 | 180 | 20599.3 | 6.14 |
| Maker00010657 | Pb10657 | Type I | Mγ | 608 | 473 | 54270.3 | 9.25 |

## Supplementary Table 13. List of reference GH3, MYB46, and EPSP genes from different species.

| **Gene family** | **soecies** | **Gene ID** | **Gene accession** |
| --- | --- | --- | --- |
| **GH3** | *Arabidopsis* | ATGH3.1 | At2g14960 |
|  | *Arabidopsis* | ATGH3.2 | At4g37390 |
|  | *Arabidopsis* | ATGH3.3 | At2g23170 |
|  | *Arabidopsis* | ATGH3.4 | At1g59500 |
|  | *Arabidopsis* | ATGH3.5 | At4g27260 |
|  | *Arabidopsis* | ATGH3.6 | At5g54510 |
|  | *Arabidopsis* | ATGH3.7 | AT1g23160 |
|  | *Arabidopsis* | ATGH3.8 | At5g51470 |
|  | *Arabidopsis* | ATGH3.9 | At2g47750 |
|  | *Arabidopsis* | ATGH3.10 | At4g03400 |
|  | *Arabidopsis* | ATGH3.11 | At2g46370 |
|  | *Arabidopsis* | ATGH3.12 | At5g13320 |
|  | *Arabidopsis* | ATGH3.13 | At5g13350 |
|  | *Arabidopsis* | ATGH3.14 | At5g13360 |
|  | *Arabidopsis* | ATGH3.15 | At5g13370 |
|  | *Arabidopsis* | ATGH3.16 | At5g13380 |
|  | *Arabidopsis* | ATGH3.17 | At1g28130 |
|  | *Arabidopsis* | ATGH3.18 | At1g48670 |
|  | *Arabidopsis* | ATGH3.19 | At1g48660 |
| **MYB46** | *Arabidopsis* | ATMYB46 | BT002549.1 |
|  | *Arabidopsis* | ATMYB83 | At3g08500 |
|  | *Arabidopsis* | ATMYB58 | At1g16490 |
|  | *Arabidopsis* | ATMYB63 | At1g79180 |
|  | *Populus trichocarpa* | PtrMYB2 | KF148677.1 |
|  | *Populus trichocarpa* | PtrMYB3 | KF148675.1 |
|  | *Populus trichocarpa* | PtrMYB20 | KF148676.1 |
|  | *Populus trichocarpa* | PtrMYB21 | KF148678.1 |
|  | *Vitis vinifera* | VvMYB46A | XP_002282821.1 |
|  | *Vitis vinifera* | VvMYB46B | XP_002282821.1 |
|  | *Glycine max* | GmMYB46A | XP_003555035.2 |
|  | *Glycine max* | GmMYB46B | XP_003539482.1 |
|  | *Glycine max* | GmMYB46D | XP_003543900.2 |
|  | *Glycine max* | GmMYB46E | XP_003555035.2 |
|  | *Eucalyptus grandis* | EgMYB2 | AJ576023.1 |
|  | *Brachypodium distachyon* | BdMYB46 | XP_003575963.1 |
|  | *Hordeum vulgare* | HvMYB46 | AAU43823.1 |
|  | *Oryza sativa* | OsMYB46 | JN634084.1 |
|  | *Zea mays* | ZmMYB46 | JN634085.1 |
|  | *Sorghum bicolor* | SbMYB46 | XP_002443268.1 |
|  | *Medicago truncatula* | MtMYB46 | XP_003597423.1 |
|  | *Pinus taeda* | PtMYB4 | AY356371.1 |
| **EPSP** | *Arabidopsis* | ATEPSP | At2g45300 |
|  | *Populus trichocarpa* | PtrEPSP1 | PNT49720.1 |
|  | *Populus trichocarpa* | PtrEPSP2 | PNT03392.1 |
|  | *Eutrema salsugineum* | EsEPSP | ESQ39162.1 |
|  | *Citrus clementina* | CrEPSP | EOA26917.1 |
|  | *Vitis vinifera* | VvEPSP | CBI38903.3 |
|  | *Theobroma cacao* | TcEPSP | EOX96244.1 |
|  | *Citrus clementina* | CcEPSP | ESR58516.1 |
|  | *Glycine max* | GmEPSP | KRH65317.1 |
|  | *Medicago truncatula* | MtEPSP | AES87336.1 |
